# Longitudinal recovery and self-efficacy in first-episode schizophrenia: insights from a 10-year follow-up study

**DOI:** 10.3389/fpsyt.2025.1588349

**Published:** 2025-06-16

**Authors:** Anne-Kari Torgalsbøen, Christine Mohn, Frank Larøi, Nikolai Czajkowski

**Affiliations:** ^1^ Department of Psychology University of Oslo, Oslo, Norway; ^2^ National Centre for Suicide Prevention and Research, Institute of Clinical Medicine, University of Oslo, Oslo, Norway; ^3^ Psychology and Neuroscience of Cognition Research Unit, University of Liège, Liège, Belgium; ^4^ Division of Mental Health, Norwegian Institute of Public Health, Oslo, Norway

**Keywords:** self-efficacy, prognosis, clinical recovery, longitudinal, first-episode schizophrenia, outcome, course

## Abstract

**Background:**

Research on clinical recovery rates in first-episode schizophrenia has yielded inconsistent results due to varying definitions of recovery and methodological differences. The longitudinal trajectory of recovery—whether rates improve, decline, or remain stable—remains unclear. Schizophrenia significantly impacts young lives, making it crucial to examine self-efficacy, the belief in one’s ability to manage adversity, and its relationship with clinical recovery.

**Methods:**

The Oslo Schizophrenia Recovery study`s repeated assessment design, including twelve clinical evaluations over ten years, is ideal for studying longitudinal recovery. Self-efficacy was measured using the General Perceived Self-Efficacy scale, with data analyzed through linear multilevel models. Twenty-eight well-defined first-episode schizophrenia patients were assessed yearly, using a strict recovery definition (two years of full symptom remission and adequate social/role functioning), with 79% of patients retained from baseline.

**Results:**

Recovery rates improved and remained stable, suggesting better outcomes than previously reported. Of the participants, 50% achieved clinical recovery. Recovered individuals showed a sharp increase in self-efficacy within the first year, while non-recovered patients exhibited gradual improvement. The interaction between recovery status and time revealed distinct self-efficacy trajectories, particularly in the first post-onset year.

**Conclusions:**

A significant proportion of first-episode schizophrenia patients can achieve clinical recovery. While these positive outcomes are noteworthy, it is important to recognize that recovery paths can vary widely among individuals. Since people with schizophrenia are concerned about their chances of recovery, the results must be shared with patients and their families. While the causal relationship between self-efficacy and recovery remains unclear, they likely influence each other.

## 1 Introduction

Numerous longitudinal studies and meta-analyses (1–7) have shown that schizophrenia is not inevitably a chronic disorder and that remission and clinical recovery are possible. A recent systematic review found a 57% recovery rate among first-episode schizophrenia (FES) studies in nonclinical trials from the 21^st^ century (8), suggesting that many individuals with FES recover. However, long-term follow-up studies (10–20 years) report clinical recovery rates between 14% to 35.2% (9–12) These variations likely arise from methodological differences, particularly the lack of consensus definitions of clinical recovery (11) and first-episode psychosis (13). Based on findings from longitudinal studies (1, 14–16) Liberman et al (16) suggest that clinical recovery be defined in a multifaceted manner that includes not only The Remission in Schizophrenia Working Group’s (RSWG) consensus definition of symptomatic remission (17), but also encompasses other aspects such as functional and social dimensions. In addition, they suggested that the required duration of recovery should be two years, with adequate functioning including at least part-time employment.

Beyond recovery definitions, patient attrition in longitudinal studies can also influence clinical recovery rates. High attrition, often due to long gaps between assessments, may affect the validity of longitudinal studies, which need a minimum retention rate of 70-80% to preserve internal and external validity (18). Studies with retention rates between 38% and 61% (9–12) may have altered recovery rates if dropout is related to the degree of recovery.

The construct of self-efficacy involves an optimistic self-belief that one can manage novel or difficult tasks and handle life’s adversities (19). Differences in perceived self-efficacy result in different ways of feeling, thinking, and acting (20). Individuals with higher levels of self-efficacy tend to approach difficult tasks as challenges to be mastered rather than to be avoided, trusting their own abilities in the face of adversity (19). In contrast, individuals with lower levels of self-efficacy tend to experience self-doubt and anxiety when they encounter environmental demands, and often shy away from such difficult situations (21).

Individuals with schizophrenia often exhibit low perceived self-efficacy compared to healthy controls (22, 23). This lower self-efficacy is associated with poorer psychosocial functioning and well-being. Conversely, higher self-efficacy levels correlate with better symptom management and interpersonal behaviors (21). Additionally, Cardenas and collaborators (24) found that motivational processes like self-efficacy help explain why some individuals with the capacity to function well do not translate this capacity into real-world functioning. Studies also highlight self-efficacy as a mediator between internalized stigma and recovery (25, 26). Importantly, self-efficacy is dynamic, and changes over time with environmental conditions, offering opportunities for intervention (20, 27). Thus, self-efficacy could be a key target for improving motivational deficits and functional outcomes in early-stage schizophrenia (28). Despite its significance, most research on self-efficacy has been cross-sectional, and focused on older and multi-episode schizophrenia patients, leaving its role in clinical long-term outcomes of FES unclear.

The Oslo Schizophrenia Recovery study (OSR) (4, 29, 30) seeks to clarify the clinical outcome of FES and elucidate the trajectories of self-efficacy over time while at the same time addressing previous methodological shortcomings. The OSR restricts FES participants to those referred within five months of their first hospitalization or outpatient clinic contact for psychosis, enabling early clinical assessment. A repeated measures design with annual assessments across 12 time points over 10 years allows for a systematic determination of when participants meet criteria for remission and clinical recovery, as well as tracking the development of self-efficacy. This approach facilitates the exploration of various aspects of the clinical course, including whether the proportion of individuals who recover increases, declines, or remains sustained over time.

In previous reports (30) from this study, 16% were clinically recovered at 2-year follow-up. This rate rose to 55% at 4-year follow-up, with 16% of the participants showing a sustained clinical recovery from year 2 (4). To fully map the clinical recovery of the sample and investigate any changes in the proportion of recovered individuals over the long term, we now extend the assessment period. To the best of our knowledge, very few studies have followed FES so closely with regular time point assessments across 10 years. The present study aims to answer the following research questions:

1. Does the proportion of clinically recovered individuals increase, decline, or sustain over a 10-year period?
2. Are there significant differences in self-efficacy development between clinically recovered and non-recovered participants?

## 2 Methods

### 2.1 Participants

Thirty-one FES patients were referred to this study during a period of 4 years (2007-2011) from various mental health service institutions in the Vestre Viken Hospital Trust (VVHF) and the Oslo metropolitan area, the majority coming from units specializing in early intervention for psychosis. This catchment area consists of rural areas as well as city centers and provides state-funded healthcare to the southeastern region of Norway. Patients were treated with psychoeducation, supportive psychotherapy, cognitive behavior therapy, antipsychotic medication, and case-management. Twenty-eight out of the 31 referred participants fulfilled the following inclusion criteria: age >18 years, the first episode of mental illness was within the spectrum of DSM-IV (31) schizophrenia and referral occurred within the first five months of their first contact with a hospital or outpatient clinic due to a psychosis. Exclusion criteria were diagnosis of an affective disorder, IQ <70 and head trauma. The sample represents about 60% of the incidence cases from the catchment area and is thus considered representative of FES in this area. After describing the study and the procedures involved, written informed consent was obtained from all participants. The study was approved by the Regional Committee for Research Ethics (REK, ref. no 2007/1139) and the research was conducted in accordance with the Declaration of Helsinki.

**Figure 1:**
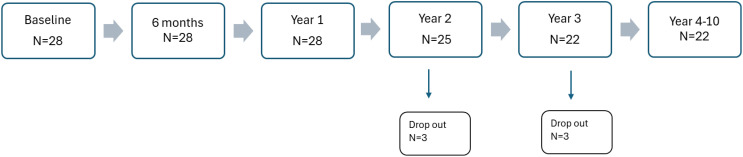
Patient flow chart across 10 years.

Participants underwent 12 assessments over ten years: baseline, six months, one year, and annually for nine more years. Three participants dropped out during the 2-year follow-up, and an additional three participants dropped out during the 3-year follow-up. Dropout reasons included anxiety, lack of awareness of mental illness, and perceived lack of research usefulness. One subject did not provide a reason, and another was untraceable. The only significant baseline difference at the 10-year follow-up was higher scores for non-completers on positive (p=0.008) and general symptoms (p=0.023) (Positive and Negative Syndrome Scale (PANSS). Twenty-two of the initial 28 were available at the 10-year follow up, resulting in a retention rate of 79%. The participant flow is shown in **Figure 1**. **Table 1** presents the demographic and clinical characteristics of the 28 participants at both baseline and the 10-year follow-up. It specifically reports on those participants who meet the criteria for a schizophrenia diagnosis at the 10-year follow-up.

**Table 1:** Demographic and clinical characteristics of the participants.

Demographic		
Age	Mean = 21.0 (SD = 2.6) 18-27 yrs.	
**Gender**		
Women	39.3% (n=11)	
Men	60.7% (n=17)	
**Level of education**		
Elementary school	39.3% (n=11)	
High school	28.6% (n=8)	
Some college	25.5% (n=7)	
BA or higher	7.2% (n=2)	
Duration of untreated psychosis	15.9 (15.4) months 1-60 months	

	Baseline (n=28)	No. of fully remitted subjects at 10-year follow up
**Clinical**		
Diagnosis		
Schizophrenia	21	
Schizoaffective	6	
Psychosis NOS	1	
Schizophrenia, single episode in full remission		2
Schizophrenia, other or unspecified pattern		12
On medication	92.8% (n=26)	68.2% (n=15)
Substance abuse, current	3.6% (n=1)	9.1% (n=2)
Substance abuse, previous	64.3% (n=18)	
**SCI-PANSS**		
Positive	18.3 (5.4) 8-30	8.1 (2.3) 7-16
Negative	20.7 (4.3) 13-31	8.6 (2.1) 7-13
General	40.2 (9.3) 22-54	19.9 (3.9) 16-30
**Global function**		
Social	6.1 (1.2) 3-8	7.1 (1.2) 4-9
Role	4.1 (1.9) 2-7	6.8 (1.4) 4-8
Hospitalized	57.0% (n=16)	
Outpatient	43.0% (n=12)	
Clinically recovered	None	50% (n=14)
In remission		71%(n=20)

Numbers in mean (SD) unless otherwise specified. Second line of cell is min-max scores. Medication: antipsychotic, mood stabilizing, and/or antidepressants.

### 2.2 Clinical instruments

Comprehensive clinical interviews specifically developed for the OSR study and self-report assessments of self-efficacy of the participants were conducted within the first five months of their admission to the hospital or outpatient clinic. Diagnoses were first made by the patients’ treating clinicians, then were separately confirmed by an experienced clinical psychologist affiliated with the study. Diagnoses were based on the Structured Clinical Interview for DSM-IV Axis I disorders (SCID-I), modules A-D (32), medical records, clinical interview and the evaluation of positive and negative symptoms using The Positive and Negative Syndrome Scale (PANSS) (33). These clinical measures were also used at the 10-year follow up. Follow-up assessments were completed by a clinical psychologist trained in PANSS ratings. To establish accuracy of remission and clinical recovery judgements, we completed an inter-rater reliability assessment, which yielded satisfactory agreement between raters (4).

On every assessment point, the participants completed the assessments described below. Everyday functioning was rated based on information from the clinical interview, and measured with the Global Functioning Scale: Social (GFS: Social) and the Global Functioning Scale: Role (GFS: Role) (34). Whereas most measures of social and role functioning in psychosis research have been developed for use with chronic adult patients, the Global Functioning scales are useful and valid measures of the more subtle features often seen in first-episode patients (35). These two 10-point scales separate social from work/school functioning domains, are sensitive to changes in functioning over time, and provide brief and easy-to-use clinician ratings, while taking age and phase of illness into account. These measures are appropriate for the prospective monitoring of individuals with FES (30).

#### 2.2.1 Assessment of self-efficacy

The Norwegian version of The General Perceived Self-Efficacy Scale (GSE) was used to measure self-efficacy (36). Respondents rate ten items on a four-point Likert scale (1 = strongly disagree, 4 = strongly agree), with higher scores reflecting perceptions of higher levels of self-efficacy. The GSE scale has strong psychometric properties (37, 38). Based on a general population sample from 25 countries, a mean GSE score of 29.6 (SD 5.3) was found (39) and will be used as a point of reference to discuss the level of self-efficacy among the participants of the present study.

### 2.3 Symptom remission definition

Symptom remission was assessed based on consensus criteria (17) evaluating eight symptom groups of the PANSS: P1 (delusions), G9 (unusual thought content), P3 (hallucinatory behavior), P2 (conceptual disorganization), G5 (mannerisms and posturing), N1 (blunted affect), N4 (social and emotional withdrawal), and N6 (lack of spontaneity). Scores for all these items must be mild or less (<3) on a 1–7 scale, lasting a minimum of six months.

### 2.4 Clinical recovery definition

Clinical recovery criteria combine the definition of symptom remission (17) with operational recovery criteria by Liberman and collaborators (16). Symptom remission criteria are based on evaluation of the eight symptom groups in PANSS, requiring mild or less scores (<3) for two years. Additionally, individuals must meet psychosocial functioning criteria: at least part-time ordinary (paid) work or school, independent living (without supervision by family), socializing with peers or otherwise involved in recreational activities that are age-appropriate and independent of professional supervision, and a score of eight on Global Functioning: Social and Role for two years. The calculation of rate of recovery was based on all the 28 baseline subjects in the study.

### 2.5 Data analyses

All statistical analyses were performed using IBM SPSS Statistics, Version 26.0. Growth models were fitted to estimate initial levels and change in self-efficacy over time. A major benefit of using multilevel modelling (MLM) is MLM’s ability to handle partially missing data (40). Since missing cases are estimated based on available data points, there is no need to remove participants with incomplete data (41). All available data are therefore included in the analysis, which is important given the size of our sample. Recent simulation studies have demonstrated that LMM’s can perform well even in smaller samples (42), providing a comprehensive discussion and applied demonstration of LMM’s in small-sample longitudinal treatment studies. These models are preferable to traditional approaches such as repeated-measures ANOVA, as they retain all available data, handle missingness effectively, and account for variability across both individuals and items. In the current study, the relatively high number of repeated observations per participant (12 timepoints) provides enough within-person information to reliably estimate fixed effects, even with a smaller number of level- 2 units. Furthermore, we specified a parsimonious random-effects structure (random intercepts and random (linear) slope for individuals.

A series of models to investigate the trajectory of self-reported GSE over 10 years in relation to recovery status was fitted. Models ranged in complexity, and included linear models, the polynomial models up to the third degree, and cubic splines. Due to singularity errors encountered with more complex random effects structures, we restricted all models to contain random intercepts and linear random slopes. For each model, we also tested an interaction between recovery group and time and investigated baseline differences. Model performance was assessed using the Akaike Information Criterion (AIC) (43) and the Bayesian Information Criterion (BIC). Additionally, we used ANOVA to test the significance of the interaction term. An overview of growth models is given in **Table 2**.

**Table 2:** Overview of growth models.

Model of self-efficacy trajectories		
	AIC	BIC
Linear No Interaction	1420.963	1442.327
Linear with Interaction	1420.753	1449.238
Linear with Interaction, Equal Baseline	1434.933	1452.737
Quadratic Polynomial No Interaction	1419.499	1444.424
Quadratic Polynomial with Interaction	1417.544	1453.151
Quadratic Polynomial with Interaction, Equal Baseline	1416.168	1448.214
Cubic Polynomial No Interaction	1415.567	1444.052
Cubic Polynomial with Interaction	1414.967	1457.695
**Cubic Polynomial with Interaction, Equal Baseline**	**1413.182**	**1452.349**
Cubic Spline No Interaction	1415.700	1469.111
Cubic Spline with Interaction	1417.796	1485.449
Cubic Spline with Interaction, Equal Baseline	1415.986	1480.078
Natural Spline with Interaction, Equal Baseline	1420.622	1509.639

## 3 Results

### 3.1 Remission and recovery status

**Figure 2:**
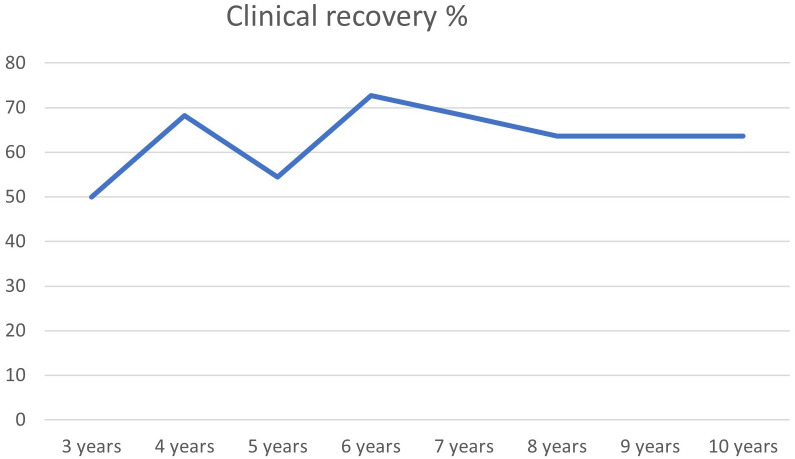
Clinical recovery 3–10 years (N=28).

At 10-year follow-up, 50% of the participants were clinically recovered. **Figure 1** shows that the proportion of recovered individuals both increase and sustain over the 10-year period. Until year 4 there is a major trend of improvement followed by some fluctuation until year 8 and thereafter stabilizing at a 50% clinical recovery rate onward to year 10. Demographic and clinical characteristics of the two groups (clinically recovered and non-recovered) at the 10-year follow-up are presented in **Table 3**. The proportion of participants reaching the criteria of clinical recovery at different assessment time points is given in **Figure 2**.

**Table 3:** Demographic and clinical characteristics of the clinically recovered and non-recovered at 10-year follow-up (n = 22).

	Clinical Recovery (n = 14)	Not clinically recovered (n = 8)
Age in years	32.0 (2.8)	31.0 (2.6)
Gender		
Female Male	57.2 % (n = 8) 42.9 % (n = 6)	25.0 % (n = 2) 75.5 % (n = 6)
In work/in education	92.9 % (n = 13)	25.0 % (n = 2)
Civil status		
In a relationship Single	57.2 (n = 8) 42.9 % (n = 6)	- 100 % (n = 8)
SCI-PANSS		
Positive	7.1 (0.3)	9.7 (3.1)
Negative	7.2 (0.8)	10.7 (1.7)
General	17.7 (1.9)	23.1 (3.9)
Global function		
Social	7.7 (0.6)	6.2 (1.3)
Role	7.8 (0.4)	5.6 (1.1)
Treatment status		
Not in psychosocial treatment	64.3 % (n = 9)	-
Outpatient	35.7% (n = 5)	75.0 % (n = 6)
Other	-	25.0 % (n = 2)
Substance abuse		25.0 % (n = 2)
Antipsychotic medication	50.0 % (n = 7)	83.5 % (n= 7)

Age, PANSS, and Global function scores in mean (SD).

### 3.2 Longitudinal modeling

To understand the trajectory of self-efficacy over time, we compared several models with increasing flexibility. The Cubic Polynomial with Interaction, Equal Baseline model provided the best fit, as indicated by the lowest AIC (1413.2) and a low BIC (1452.3) (**Table 2**).

**Figure 3:**
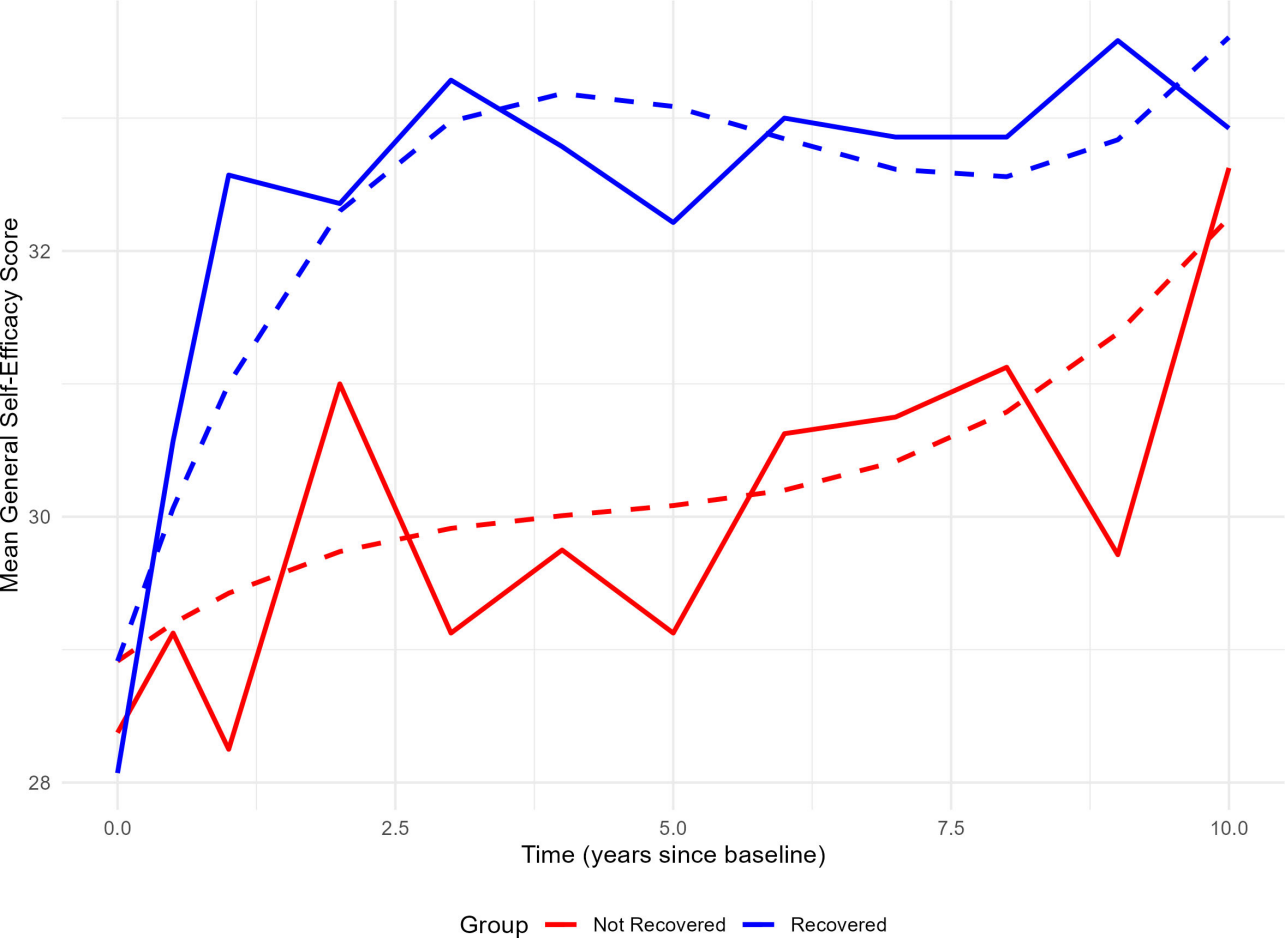
Development of self-efficacy over 10 years among recovered and non-recovered individuals. Solid lines show the observed group-level mean scores of general self-efficacy (y-axis) measured at multiple timepoints (x-axis), ranging from baseline to 10 years. Dashed lines represent predicted values from a cubic polynomial mixed-effects model with interaction between time and recovery status. The blue lines indicate individuals who were clinically recovered after 10 years, while red lines represent those who were not recovered. The model accounts for individual differences by including random slopes and intercepts for time.

An ANOVA comparison between the cubic model without interaction (AIC = 1415.6, BIC = 1444.0) and the cubic model with interaction (AIC = 1413.2, BIC = 1452.3) showed that the interaction term was significant (p = 0.039). The significant interaction between time and recovery status revealed distinct trajectories for the two groups. Individuals in the clinically recovered group experienced a steep increase in self-efficacy during the first year, whereas those who did not recover showed a more gradual increase throughout the entire period (**Figure 3**).

## 4 Discussion

The OSR study is the first to employ a repeated assessment prospective design on a well-defined sample of individuals with First Episode Schizophrenia (FES) over a 10-year period, and furthermore achieving a low dropout rate. Our findings reveal a high rate of strictly defined clinical recovery at 50%, which aligns with the results from Huxley’s meta-analysis (2021). These findings challenge some views about the long-term outcomes for individuals diagnosed with FES. First, our results indicate that a substantial proportion of FES patients can achieve clinical recovery. The progression towards clinical recovery was gradual, with some participants achieving recovery earlier in the study period while for others it took longer to reach this level of recovery. This variation in clinical recovery timelines underscores the individualized nature of the recovery process in FES. Second, the detailed tracking of the same subject’s recovery status across 10 years revealed that for a majority, the recovery was sustained during the follow-up years. Finally, for a subgroup, clinical recovery was achieved early in the course and sustained over time.

Comparing these rates of clinical recovery with results from other studies is difficult due to significant differences in study characteristics such as age at intake, study design, and in the definitions of clinical recovery and the sample. A recent longitudinal study from Norway (11) highlights these differences. They reported a clinical recovery rate among FES patients of 22.9% at 10-year follow-up, but the sample was defined differently (i.e., first-episode psychosis was defined as recruitment within the first 52 weeks after the start of first adequate treatment), the participants were older at intake (mean age 26.9), and they were only assessed twice during the 10-year period (at baseline and 10 years later). Furthermore, the retention rate was low (38.1%). Such a design (cross sectional assessment at two points) is less sensitive to changes that occur between the assessment points and furthermore makes it more difficult to keep the participants in the study.

Limitations associated with research design, coupled with potential selection bias from the dropout of individuals with good recoveries, likely contribute to an underestimation of clinical recovery rates for FES in the existing literature. A meta-analysis and systematic review conducted by Lally et al (7) indicates that higher dropout rates may be associated with lower recovery levels, suggesting a selection bias wherein individuals who have improved and are no longer in contact with mental health services may be disproportionately lost to follow-up, thus affecting the perceived recovery rate. In a similar vein, Simonsen et al (44) argue that attrition in their longitudinal study could be attributed to very poor outcome (too symptomatic) or very good outcome (too busy with work). Furthermore, a publication bias, indicated by a relative scarcity of studies (especially small studies) reporting high recovery rates, suggests an underestimation of the true recovery rate (45). This situation underscores the importance of conducting further research to accurately assess recovery outcomes for individuals experiencing first-episode schizophrenia.

The high retention rate in our study, with 22 out of 28 patients completing all the interviews and assessments from year 4 and onward to the 10-year follow-up, may contribute to our percentage of clinically recovered participants, Furthermore, our study design included stable timepoints (1 year between assessments) and multilevel analyses. This provides the unique and crucial possibility to capture both changes and stability in the same individuals over time, demonstrating that a majority moved towards health, albeit at different timepoints during the course.

In addition to the design of the OSR study, one factor that might contribute to explain some of the discrepancy in clinical recovery rates compared to other longitudinal studies is the education level of our sample. 33% have an education level beyond senior high school. Completing high school suggests these individuals were less affected by the illness during critical social, educational, and vocational milestones. Therefore, they may have a stronger foundation for regaining previous levels of functioning once symptoms subside. In a previous report from the OSR study (30), we showed that years of education was a significant predictor of role functioning 2 years later, indicating that good premorbid adjustment is associated with good outcome. It is also noteworthy that 21.4% of the sample are diagnosed with schizoaffective disorder, a group that appears to have a slightly better prognosis (46).

Consistent with prior research, our findings indicate that a significant number of individuals with FES experience symptom remission within the initial year of illness (9) (47, 48). For some, this remission leads to sustained clinical recovery, even without the use of continued antipsychotic medication (49). Notably, at the 10-year follow-up, 32% of the total sample were not using antipsychotic medication, which is consistent with evidence that there exists a subgroup of FES (20-40%) who can achieve recovery after several years without the need for continued antipsychotic medication (49, 50).

The findings related to our second research question revealed that the development of self-efficacy was distinct in the two groups of clinically recovered and non-recovered participants. Self-efficacy showed a very steep increase early in the course for the group that recovered, and a more gradual increase for the non-recovered. In fact, the level of self-efficacy at 10 years was almost equivalent in the clinically recovered and non-recovered groups. One possible interpretation is that achieving clinical recovery influences self-efficacy, which in turn has a self-reinforcing effect on further development. The non-recovered group also showed improved self-efficacy, but this occurred more gradually. However, self-efficacy does not necessarily follow the same trajectory for those who recover and those who do not. The almost equivalent level of self-efficacy at the 10-year follow-up between the two groups suggests that motivational processes (i.e., self-efficacy) might shed some light on why some individuals have the capacity to function well, but do not necessarily translate this capacity into real-world functioning (the non-recovered group).

According to the self-efficacy theory (19), performing in real-world circumstances is a function of having both the skills and the self-belief that one can utilize them. Nevertheless, it is interesting that even those who had not recovered still had a belief that they had the capacity to function well, probably contributing to the presence of positive mental health despite remaining mental illness and lack of adequate functioning (51). On the other hand, these results might also suggest that the non-recovered group gradually develops more belief in their abilities, but perhaps it is their lack of skills as well as their lack of insight that explains why they do not function well.

The steep improvement in self-efficacy in the clinically recovered group might also be influenced by environmental factors such as access to vocational and social participation in Norway. At the 10-year follow up, most participants in the clinically recovered group were working full-time or part-time in regular jobs, which builds self-efficacy by having an income and feeling competent. One might argue that the non-recovered participants have had the same access to work opportunities, but due to a lack of belief in using their skills, they are not in regular jobs. But we also must consider that a poor outcome group was identified early in the course of illness in this study (52). This subgroup had the largest cognitive impairments at the onset of the disorder and may have special rehabilitation needs. It is likely that greater skills development leads to a greater and more accurate level of belief in one’s skills. Thus, building self-efficacy without simultaneously increasing skills (e.g., through cognitive training or social skills training) may increase motivation to improve without providing the skills to improve functioning. This lack of skills may prevent them from achieving regular jobs and establishing a relationship, which was the case for 29% of the clinically recovered in this study.

For the clinically recovered, self-efficacy may be even more strongly experienced through independence, employment, and freedom from psychosis. Individuals who have clinically recovered may attribute their improvement to their own efforts rather than external factors, such as the passage of time. This attribution may boost their self-efficacy. Consequently, it is plausible that the experience of being in symptom remission and fully recovering from the first episode of schizophrenia has shaped their attitudes, potentially impacting their scores on the self-efficacy scale.

Our study’s main strength is its 10-year repeated assessments design, ensuring high retention in a well-defined FES sample. This highlights the importance of effective follow-up systems and participant satisfaction. The high retention rate enabled robust multi-level analyses. It is important to emphasize that our study included clinically recovered participants who, during the follow-up period, had discontinued treatment and were not in contact with mental health services. As suggested by Lally et al (7), patients who are doing well may be lost to follow-up, which could impact the overall recovery rates. This hypothesis warrants further exploration. Additionally, our stringent and explicit definition of clinical recovery (rated at 80 out of 100 in a review) (45) facilitates replication in larger samples. Lastly, our focus on including only the “true” first episodes, in a country with a public mental healthcare system, supports the representative nature of our sample, even if it is small.

### 4.1 Limitations

A nostable limitation of this study is the small sample size, which restricts the generalizability of our findings. However, acquiring a larger sample is challenging in longitudinal studies that, in addition, involve numerous repeated assessments. While the dropout rate was relatively low (21%), the non-completers exhibited significantly higher positive and general symptomatology than the completers, potentially affecting the recovery rates. Therefore, caution is advised when interpreting our findings for FES patients in other countries until they are replicated in larger samples using similar methodologies. Additionally, we cannot ascertain the causal relationship between changes in self-efficacy and recovery over time, although we suspect that they influence each other. Our findings have important clinical implications. With 46% of participants being employed or in education along with 29% being in a relationship, these results suggest a more positive outlook for FES than earlier reported in a meta-analysis by Ajnakina et al (53) (32.5% and 21.3% respectively). Integrating self-efficacy into clinical assessments and targeted interventions can enhance personal competence, especially in the early course of illness.

## Data Availability

The raw data supporting the conclusions of this article will be made available by the authors, without undue reservation.
